# What can fruit flies teach us about karate?

**DOI:** 10.7554/eLife.04040

**Published:** 2014-08-19

**Authors:** Helen H Yang, Thomas R Clandinin

**Affiliations:** 1 **Helen H Yang** is in the Department of Neurobiology, Stanford University, Stanford, United States; 2 **Thomas R Clandinin** is in the Department of Neurobiology, Stanford University, Stanford, United Statestrc@stanford.edu

**Keywords:** serial behavior, grooming sequence, action selection, behavioral choice, competitive queuing, competing motor programs, *D. melanogaster*

## Abstract

Understanding the logic behind how a fruit fly's brain tells it to groom its body parts in a stereotyped order might help us understand other behaviours that also involve a series of actions.

**Related research article** Seeds AM, Ravbar P, Chung P, Hampel S, Midgley Jr FM, Mensh BD, and Simpson JH. A suppression hierarchy among competing motor programs drives sequential grooming in *Drosophila*. *eLife* **3**:e02951. doi: 10.7554/eLife.02951

**Image** Fruit flies groom themselves to remove dirt and dust from their bodies

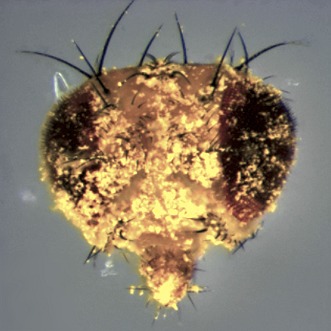

When performing kata, a skilled martial artist flows through a sequence of individual movements—attacks and blocks, turns and jumps—to defeat a series of imaginary opponents. Of course, such action sequences are not restricted to either karate or humans. Instead, serial behaviours are widespread throughout the animal kingdom, with examples including navigation, courtship and communication. However, it is not clear how a nervous system is able to generate sequences of actions.

Now in *eLife*, Andrew Seeds and co-workers—who are all at the Janelia Farm Research Campus—have taken a novel approach to investigate serial behaviours by using the powerful genetic tools that are available in the fruit fly *Drosophila* (Seeds et al., 2014). Two different procedures, or algorithms, have previously been proposed for the production of sequential actions (Houghton and Hartley, 1995). ‘Response chaining’—in which each action triggers the next like a falling row of dominos (Adams, 1984)—is thought to underlie birdsong (Long et al., 2010). Alternatively, all of the actions in a sequence could be prepared in parallel, with an inbuilt order of priority and a winner-take-all competition leading to one action being performed at a time and in the right order. Typing and speaking, for example, are human behaviours that are thought to be implemented through this ‘competitive queuing’ model (Lashley, 1951). However, it has been difficult to obtain causal evidence that would favour one of these algorithms over the other. Now Seeds et al. demonstrate that competitive queuing produces the sequential grooming behaviour observed in fruit flies.

Grooming is a patterned, or stereotyped, sequence of cleaning movements performed by most animals with limbs. Indeed, fruit flies use their front or hind legs to clean specific body parts when they are dirty (Szebenyi, 1969). Seeds et al. completely covered flies with dust and observed that each fly preferentially cleaned its eyes first, followed by its antennae, its abdomen, its wings, and finally its thorax. These observations reveal two points: first, grooming in dirty fruit flies is a serial behaviour; and second, it follows a stereotyped order.

So, how does the fruit fly's nervous system produce this serial behaviour? To address this question, Seeds et al. first identified populations of neurons that, when activated, were able to trigger grooming of each body part in the absence of dust. This revealed that at the neuronal level as well as at the behavioural level, grooming is comprised of individual, separable units or modules. Then, rather than asking which neurons are involved in the cleaning of each body part, Seeds et al. took a novel approach towards understanding the processes, or computations, that underpin grooming and instead asked: what algorithm does the brain use to generate this sequence?

If response chaining were used to control this behaviour, grooming of one body part would trigger the grooming of the next one in the sequence. However, this was not the case. Instead, Seeds et al. show that it is the stimulus—the presence of dust—that drives cleaning of each body part. The cleaning of different body parts, however, is not only affected by dust, since the grooming of one body part blocks the grooming of body parts that follow later in the cleaning sequence. For example, when Seeds et al. covered a fly with dust and activated its abdomen-grooming module at the same time, the fly cleaned its eyes, antennae and abdomen, but never cleaned its wings and thorax. When eye cleaning was activated, the flies continuously groomed their eyes even though there was dust covering the rest of their bodies too. This demonstrates that the order in which body parts are groomed reflects an underlying set of priorities. That is, higher priority behaviours (such as eye cleaning) suppress lower priority behaviours (such as abdomen cleaning), but not vice versa. Thus, these modules interact in a ‘suppression hierarchy’.

The results indicate competitive queuing, and Seeds et al. used a simulation to demonstrate that such a model is sufficient to reproduce sequential grooming behaviour (Figure 1). In a dirty fly, all of the cleaning modules are activated in parallel, but they are prioritized by a suppression hierarchy; a winner-take-all competition then selects the highest priority module, and this module is performed. Once dust is cleaned from that body part, the stimulation of the corresponding module is also removed, and the next most important module is performed.

**Figure 1.**
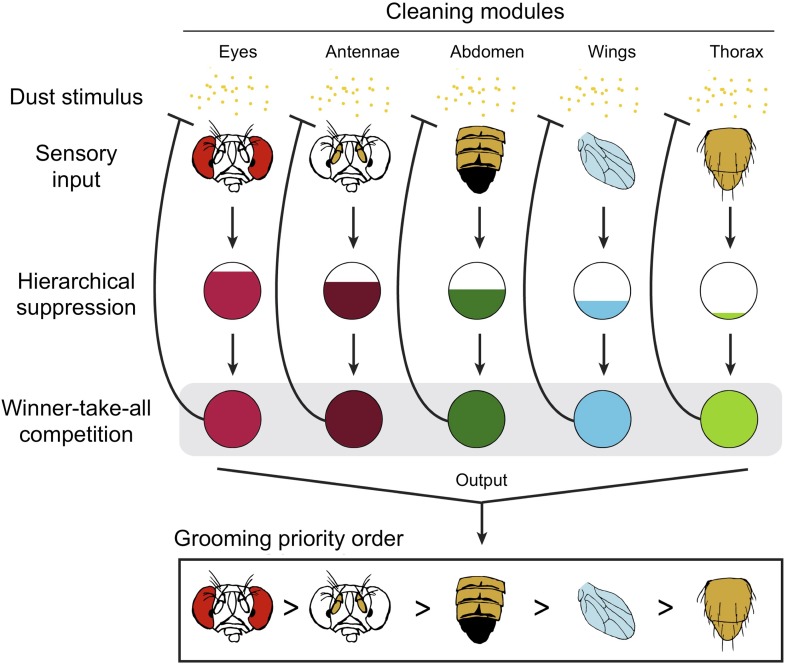
Competitive queuing produces sequential grooming behaviour. Seeds et al. suggest that grooming behaviour in fruit flies uses a three stage algorithm to select which cleaning module to perform: sensory input (top), hierarchical suppression (middle), and a winner-take-all competition (bottom). The dust (yellow dots) activates each cleaning module. When the fly is completely covered with dust, all the modules receive sensory input and are activated in parallel. The ‘hierarchical suppression’ stage determines the degree of activation for each module (represented by the area of the coloured region in each circle) in order to implement the order of priority. The most active module is selected and the corresponding body part is cleaned, with all other cleaning modules being suppressed. As dust is removed from a body part, the sensory input to that module is reduced (represented by the blunt arrow). Eventually, this module is no longer the most active module and a new module is selected. Multiple iterations of this process produce the sequential grooming behaviour observed in fruit flies.

Seeds et al. suggest two possible ways of implementing hierarchical suppression. A module could be high priority either because it is more sensitive to dust or because it inhibits the downstream, ‘low priority’, modules. However, further study is needed to determine which is the case for grooming behaviour in fruit flies. Furthermore, the cells and circuits that implement grooming behaviour also remain to be identified.

This study provides causal evidence that modules being activated in parallel combined with hierarchical suppression can produce serial behaviour. More broadly, it also illustrates the usefulness of studying model organisms, as it is remarkable that the same algorithm that is thought to underpin complex, learned behaviours unique to humans (such as typing and speech) also produces a widespread behaviour that is innate in fruit flies (i.e. grooming). Perhaps this algorithm is evolutionarily ancient and has been elaborated in humans, in which case, understanding computation in fruit flies can indeed teach us about karate.
